# Identifying the patterns of changes in α‐ and β‐diversity across *Dacrydium pectinatum* communities in Hainan Island, China

**DOI:** 10.1002/ece3.7361

**Published:** 2021-03-13

**Authors:** Haodong Liu, Hua Liu, Yongfu Chen, Zhiyang Xu, Yunchuan Dai, Qiao Chen, Yongkang Ma

**Affiliations:** ^1^ Research Institute of Forest Resource Information Techniques Chinese Academy of Forestry Beijing China; ^2^ Key Laboratory of Forest Management and Growth Modelling NFGA Beijing China; ^3^ East China Inventory and Planning Institute of National Forestry and Grassland Administration Hangzhou China; ^4^ Research Institute of Forest Ecology Environment and Protection Chinese Academy of Forestry Beijing China

**Keywords:** biodiversity hotspots, coexistence, community assembly, habitat filtering, phylogenetic diversity

## Abstract

Exploring vegetation distribution spatial patterns facilitates understanding how biodiversity addresses the potential threat of future climate variability, especially for highly diverse and threatened tropical plant communities, but few empirical studies have been performed. *Dacrydium pectinatum* is a constructive and endangered species in the tropical mountain forests of Hainan Island, China. In this study, sixty‐eight 30 m × 30 m permanent plots of *D. pectinatum* were investigated, and species‐based and phylogenetic‐based methods were used to analyze the α‐ and β‐diversity pattern variation and its key drivers. Our study showed that species and phylogenetic α‐diversity patterns are different on a local scale. However, on a regional scale, the variations in the two α‐diversity patterns tend to converge, and they decrease with increasing elevation. The phylogenetic structure changes from overdispersion to convergence with increasing elevation. Soil (SOM, TP, AP), topography (EL, SL), and stand (CD) factors and α‐diversity showed close correlations. Species and phylogenetic β‐diversity have significant positive correlations with changing environmental distance and geographical distance; however, as a representative form of habitat heterogeneity, elevation distance has a greater impact on β‐diversity changes than geographical distance. In conclusion, the α‐ and β‐diversity patterns of the *D. pectinatum* community are mainly related to habitat filtering, especially in high‐elevation areas, and the colonization history of various regions also affects the formation of diversity patterns. Species‐based and phylogenetic‐based methods robustly demonstrated the key role of the habitat filtering hypothesis in community assembly. We believe that more plant diversity patterns need to be explored to understand the biodiversity formation mechanisms in tropical forests. We also recommend strengthening the construction and management of nature reserves to help address the biodiversity loss crisis in endangered tropical plant communities.

## Graphical abstract

Changes in α‐ and β‐diversity of *Dacrydium pectinatum* communities are closely related to environmental filtration and diffusion restrictions. Species coexistence in tropical plant communities requires mild environmental conditions, and low temperature, precipitation, soil nutrients, and light will aggravate environmental filtering and species competition.

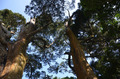

## 1 INTRODUCTION

One of the central themes in ecology is understanding the spatial patterns in biodiversity along various environmental axes (Bagchi et al., 2011). This understanding helps address the looming threats to biodiversity by contributing to regional‐scale biological conservation activities, such as reserve design and habitat restoration (Mori et al., 2013). In recent years, more than 100 hypotheses have been proposed to explain biodiversity patterns at the community level (Ricklefs, 2004). Two of the most widely discussed hypotheses pertain to niche‐based deterministic and neutrality‐based stochastic processes (Wright, 2002). In deterministic processes, limiting similarity (such as through competition, facilitation, and predation) and environmental filtering (including through energy availability, water availability, energy–water balance, and habitat heterogeneity) play key roles (Qin et al., 2017). In contrast, neutral processes emphasize the great roles of stochastic immigration and mortality in community assembly, with the actual level of species richness determined by the size of the regional species pool (Rosindell et al., 2011). Based on the assumption of dispersal limitation, the similarity between communities is expected to decrease with increasing spatial distance, and this decreasing pattern is related only to geographical distance rather than the influence of environmental factors (Tang et al., 2012).

Strong evidence indicates the existence of both significant variation and a large degree of overlap in microhabitat preferences among species in diverse subtropical and tropical forests (Kraft et al., 2008; Lai et al., 2009), and species with similar microhabitat requirements often spatially overlap with each other (Song et al., 2018). Additionally, studies have shown that plant species in communities tend to show strong competition, and microhabitat preference usually corresponds to a narrow spatial scale (Willis et al., 2010); however, changes in geography and the environment, as well as other spatial factors, have gradually enhanced the assembly effect of the community as the spatial distance has increased (Chave, 2004). Hence, the local‐scale diversity patterns addressed by niche theory might be insufficient for explaining the complex structure of tropical plant communities (Wang et al., 2016). There is a current interest in merging niche and neutral theory to explain the biogeographic patterns of diversity (Adler et al., 2007; Alonso et al., 2006), which will help eliminate some key flaws in niche theory, such as diffuse restrictions and stochastic immigration during the process of community assembly (Etienne & Alonso, 2005). Exploring the relationship between β‐diversity and environmental differences and geographical distance is an effective way to reveal how biodiversity varies along environmental axes (Chesson, 2000). The most widely recognized spatial patterns are the gradients that occur with elevation and latitude (Molina‐Venegas et al., 2019). Knowledge of these patterns is also critical for predicting how global climate change will affect future biodiversity distributions, as most species are expected to be forced to shift their distributions to higher elevations/latitudes in a warmer future (Molina‐Venegas et al., 2019; Mori et al., 2013).

Traditional species diversity emphasizes equality among species while ignoring evolutionary differences across diverse species (Graham & Fine, 2008). However, phylogenetic biology currently uses the close associations between species to speculate about the mechanisms of community assembly, which helps bridge the gap between evolutionary and community ecology (Kraft & Ackerly, 2010). Ecologists generally believe that closely phylogenetically related species are more functionally similar than distantly related species (Xu, Chen, et al., 2017; Xu, Ma et al., 2017). Nevertheless, limiting similarity might lead to phylogenetic overdispersion among coexisting species, allowing them to compete with each other for finite resources (Webb, 2000). In contrast, environmental filtering may cause the accumulation of some species with similar niche characteristics, thus forming a convergence pattern in terms of phylogenetic distance (Kraft et al., 2008). Additionally, if neutral processes drive community assembly, phylogenetic structures usually exhibit a randomized distribution (Cornwell & Ackerly, 2009). However, some researchers have come to the opposite conclusion that phylogenetic patterns may have limited use as proxies of community assembly, showing that the phylogenetic dispersion of communities is of limited value for understanding ecological assembly processes and can only be used to reveal the macroevolutionary idiosyncrasies of the habitat of their associated lineage pool (Gerhold et al., 2015). These contradictory results indicate that correctly identifying the assembly patterns of communities remains a major challenge for ecologists. Hence, to achieve a mechanistic view of the community assembly process along environmental gradients, researchers should simultaneously analyze phylogeny and traditional measures of species diversity.

Despite substantial efforts, until now, the underlying drivers of the spatial organization of diversity in tropical plant communities have remained difficult to generalize (Mokany et al., 2011). For instance, environmental filtering was found to be the main factor driving community assembly during succession in a tropical forest in New Guinea (Whitfeld et al., 2014). However, some studies have also supported the idea of incomplete filtering (Song et al., 2018), showing that geographical and environmental factors jointly contribute to the community assembly process in tropical forests (Zhang et al., 2013). Given these contrasting results, there is still much to learn about the mechanism of biodiversity formation in the tropics. Hence, clarifying the spatial distribution patterns of various diversity indicators, such as plant composition/diversity and phylogenetic structures, helps reveal the biogeographical patterns, assembly processes, and mechanisms of the formation of biodiversity. It may also provide a scientific basis for the proper management, sustainable utilization, and sound conservation of resources in different vegetation types.

**FIGURE 1:**
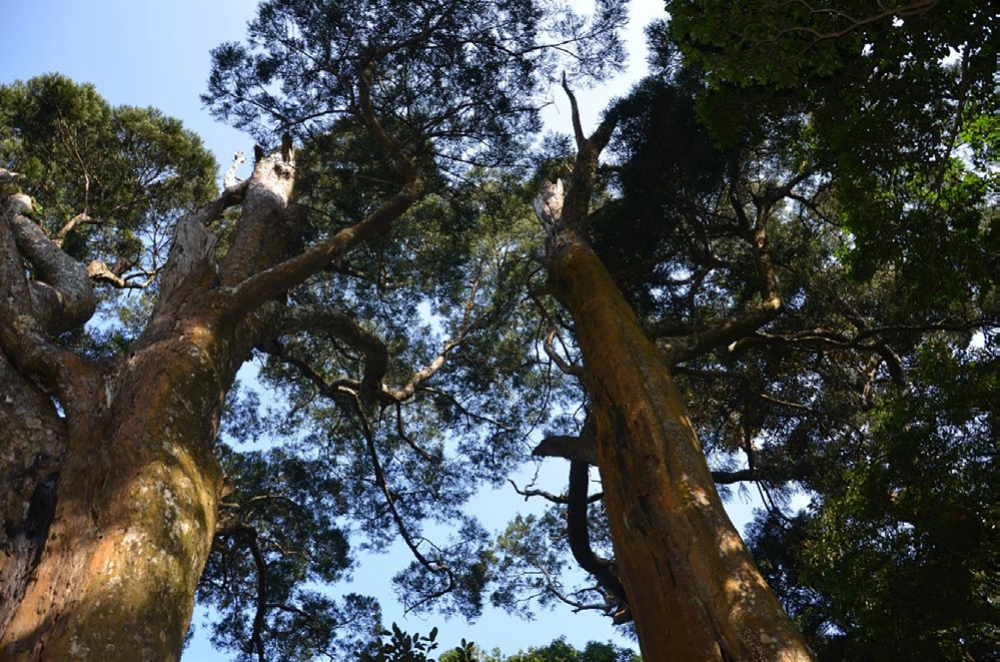
*Dacrydium pectinatum* in a tropical mountain forest on Hainan Island, China

Hainan Island in China is floristically rich and has been listed as an internationally significant biodiversity conservation area (Myers et al., 2000). Based on current records, approximately 4,600 woody plants have been documented, and 397 of these plants are endemic to the island (Francisco‐Ortega et al., 2010). *Dacrydium pectinatum* is one of the constructive and endangered species in the tropical mountain forests of Hainan Island and belongs to the Podocarpaceae family (Keppel et al., 2011). Its modern distribution in the Northern Hemisphere is limited to south of approximately 20°N, and it appears to gone extinct in Australia during the Miocene (Norton et al., 1988). *D. pectinatum* is the only species of its genus that exists in China and is found only in four original tropical rainforests on Hainan Island, namely Bawangling, Jianfengling, Diaoluoshan, and Wuzhishan (Huang et al., 2014). According to our previous investigation, the distribution range of *D. pectinatum* is usually in the mountainous area of 700–1,100 m; its maximum tree height and diameter at breast height exceed 30 m and 1.5 m, respectively (Figure 1) (Liu et al., 2020). The plant diversity of the natural community formed by this species as an absolute dominant tree species is far greater than that of tropical or subtropical forests at similar latitudes (Liu et al., 2020).

**FIGURE 2:**
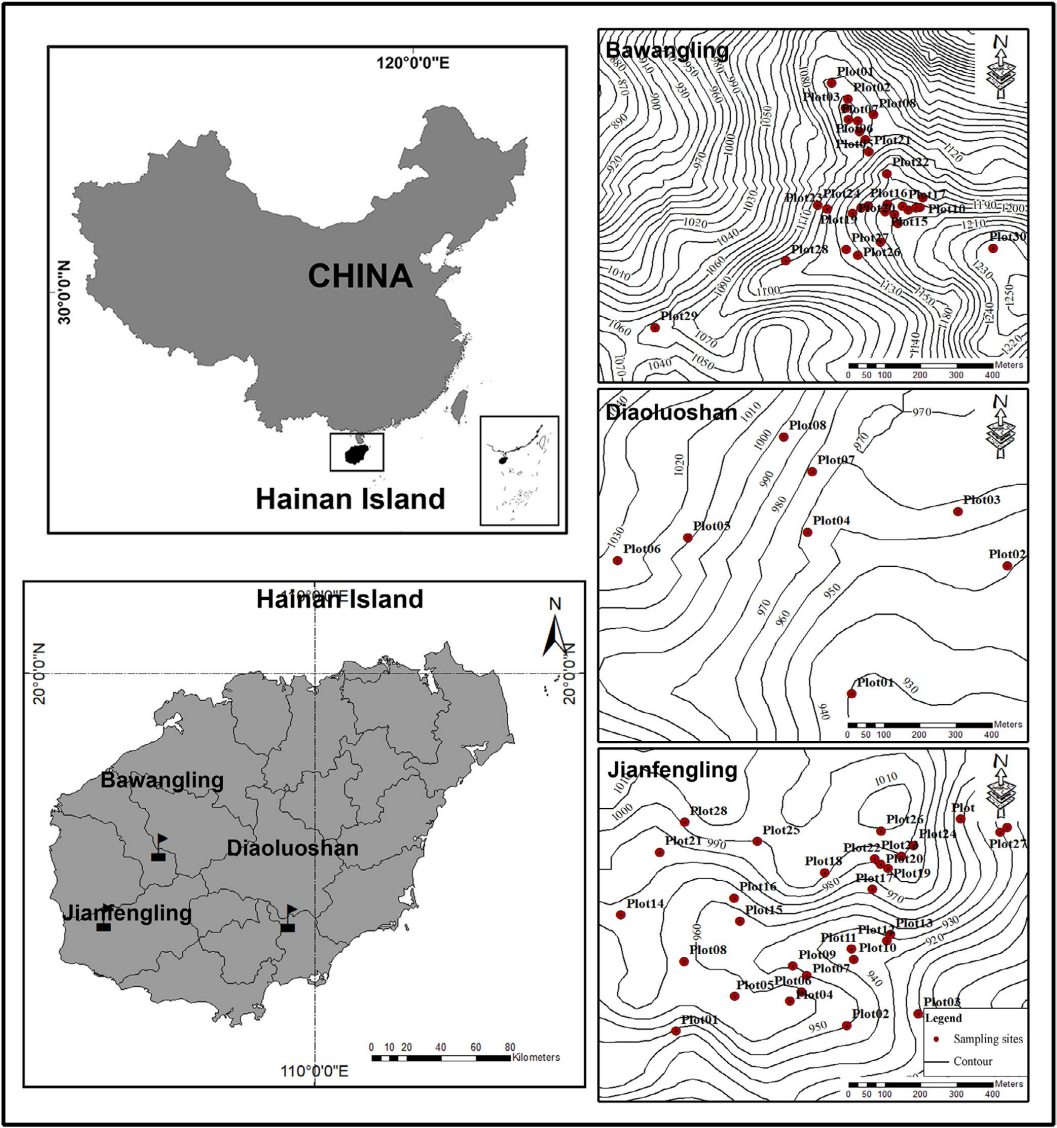
Permanent plot locations in the natural *Dacrydium pectinatum* communities in the three national nature reserves of Bawangling, Diaoluoshan, and Jianfengling, Hainan Island, China

In this manuscript, we used species‐based and phylogeny‐based methods to analyze the variation in the structure of *Dacrydium pectinatum* communities across three national nature reserves in Bawangling, Diaoluoshan, and Jianfengling on Hainan Island, China (Figure 2), which have different climatic and geographical features and are the main distribution zones of *D. pectinatum*. Specifically, our objectives were to (a) explore the distribution patterns and environmental determinants of species and phylogenetic α‐diversity at different spatial scales, (b) clarify the change patterns of species and phylogenetic β‐diversity and assess the degree to which species composition variation may be explained by elevation distance, geographical distance, and environmental gradients, and (c) understand and reveal the assembly process of the *D. pectinatum* plant community based on these findings. We expect that this study will increase our understanding of the biodiversity formation and biodiversity conservation mechanisms of tropical plant communities on Hainan Island, China.

## 2 MATERIALS AND METHODS

### 2.1 Study sites

Hainan Province is located in southeastern China (18.14°–20.02°N, 108.62°–111.05°E) at the northern edge of tropical Asia (Figure 2); its total land area is 35,400 km^2^, of which Hainan Island accounts for 33,900 km^2^, and the sea area is approximately 2 million km^2^. The island features high elevations in the middle and low elevations on all sides. The average annual precipitation is 923–2459 mm and decreases from east to west, and the annual average temperature is 22.5–25.6°C and decreases from south to north. The southwest and west are affected by ocean monsoons, and there are obvious wet and dry seasons. More clouds and lower temperatures occur in the central mountainous region than elsewhere on the island. The current study was conducted in Bawangling, which is located in the southwestern part of Hainan Island (18°53′–19°20′N, 108°58′–109°53′E); Diaoluoshan, which is situated in the southeastern part of Hainan Island (18°43′–18°58′N, 109°43′–110°03′E); and Jianfengling, which is located in the southwestern part of Hainan Island (18°23′–18°50′N, 108°36′–109°05′E). These areas are national nature reserves in China (Figure 2).

The total area of the Diaoluoshan Nature Reserve is approximately 37,900 hm^2^, and it was established in 1994. The reserve has a marine tropical monsoon climate. The annual mean temperature is 24.4°C, and the annual mean precipitation is 1,870–2,760 mm. The topography ranges from high elevations in the northwest and low elevations in the southeast, ranging between 50 and 1,499 m. The soil types are mainly mountain yellow soil and mountain red soil. The forest vegetation types in the region are mainly tropical secondary forest, tropical monsoon forest, and tropical evergreen broad‐leaved forest, among which 1955 species of vascular plants in 194 families and 870 genera have been recorded (Wang et al., 2007).

The total area of the Bawangling Nature Reserve is approximately 72,000 hm^2^, and it was established in 1980. The reserve has a tropical monsoon climate. The annual mean temperature is 23.6°C, the annual mean precipitation is 1,500–2,000 mm, and the average relative humidity is 65%–90%. The topography is mainly mountainous, and the elevation ranges between 100 and 1,654 m. The soil type is mainly brick‐red soil formed from granite and sandstone. The vegetation types are mainly low mountain rainforest, gully rainforest, and mountain rainforest, among which 2,213 species of vascular plants in 220 families and 967 genera have been recorded (Long et al., 2011).

The total area of the Jianfengling Nature Reserve is approximately 640 km^2^, and it was established in 1960. The reserve has a monsoon climate of tropical islands at low latitudes. The annual average temperature decreases from 25 to 17–19°C, and the annual precipitation increases from 1,300 to 3,500 mm along a horizontal distance of approximately 15 km from the coast to the highest elevations of the forest hinterland (the elevation ranges from 200 to 1,412 m). The coastal areas are dominated by coastal sandy soil, dry red soil, brick‐red soil, and brick‐yellow soil. The highest mountain areas mainly have leached‐surface latent yellow soil. The vegetation types in the region include tropical semideciduous monsoon rainforest, tropical evergreen monsoon rainforest, coastal barbed scrub, savanna, and dwarf moss forest on the top of the mountain, among which 2,258 species of vascular plants have been recorded (Xu et al., 2013).

The detailed coordinate, climate, and topography data for the three study sites are shown in Table 1 (elevation data from field records). The climate data, including the annual mean temperature and the annual precipitation, were extracted from the China Meteorological Data Network of the China Meteorological Administration Meteorological Data Center http://data.cma.cn/en.

**TABLE 1:** Topographic and climatic characteristics across the three *Dacrydium pectinatum* communities

Forest type	Latitude	Longitude	Elevation	Temperature	Precipitation
BWL					
TMRF	18°57′N	109°03′E	1,155.78 ± 56.93^b^	20.74 ± 1.78^a^	1617.76 ± 116.52^b^
DLS					
TMRF	18°43′N	109°43′E	935.97 ± 22.96^a^	21.89 ± 1.60^b^	1806.73 ± 98.13^c^
JFL					
TMRF	18°20′N	108°41′E	907.43 ± 46.54^a^	21.75 ± 1.52^b^	1,520.33 ± 135.14^a^

Note

Data with different letters (a, b, and c) are significantly different at *p* < .05; TMRF represents tropical mountain rainforest. BWL represents Bawangling, DLS represents Diaoluoshan, and JFL represents Jianfengling. Elevation (m), temperature (°C), and precipitation (mm) are expressed as the mean ± *SD*.

### 2.2 Field investigation and plant sampling

Field investigations were conducted from December 2017 to July 2018. Because endangered species mostly occur in low‐density populations with small quantities and scattered distributions, the minimum‐area sampling method was more suitable for this study than the large‐scale survey method. We established sixty‐eight permanent plots across Bawangling, Diaoluoshan, and Jianfengling. Based on previous surveys and the experience of local forestry workers, we constructed the plots under the following considerations: (a) the plots must contain varying numbers of adult *D. pectinatum* individuals, and they must be the absolute dominant tree species in the community; (b) the conditions within the stand must be relatively uniform and show limited human interference; and (c) if the sites meet the above criteria, a plot was randomly established within the stand. We established 30 plots in Bawangling and 30 plots in Jianfengling, each of which had an area of 900 m^2^ (30 × 30 m). Due to the severe deforestation in Diaoluoshan, we established only 8 permanent plots at this location.

All woody stems (including those of trees, shrubs, and lianas) with a diameter at breast height (DBH) ≥5 cm were identified to the species level, tagged, and mapped, and their DBH was measured. The nomenclature follows that used in the Flora of China (http://foc.eflora.cn/). Their health status was recorded (such as living trees, dying trees, and dead trees; note that only living trees were quantitatively analyzed in this study), and their height, crown width, branch height, and spatial position were measured. In each plot, three twenty‐five m^2^ (5 × 5 m) subplots were also established, and woody stems with a DBH < 5 cm were recorded by referring to the above criteria.

In total, 52 families, 101 genera, 187 plant species, and 5,386 stems; 42 families, 73 genera, 126 plant species, and 1,023 stems; and 54 families, 110 genera, 186 plant species, and 5,705 stems were recorded in Bawangling, Diaoluoshan, and Jianfengling, respectively (Table 2). The number of Lauraceae plants was most abundant, with totals of 10 genera and 24 plant species, 10 genera, and 16 plant species, and 8 genera and 25 plant species in Bawangling, Diaoluoshan, and Jianfengling, respectively (see Appendix S1). In addition, we listed the top ten species in terms of importance values (IVs). The IV is the sum of the relative density, relative frequency, and relative dominance of a species in the community, and its numerical range is generally between 0 and 300; it represents the relative importance of a given species in the community. Only 7.69% of the families (*Podocarpaceae*, *Fagaceae*, *Magnoliaceae*, and *Polygalaceae*), 14.28% of the families (*Podocarpaceae*, *Fagaceae*, *Hamamelidaceae*, *Theaceae*, *Magnoliaceae*, and *Polygalaceae*), and 7.41% of the families (*Ulmaceae*, *Palmae*, *Podocarpaceae*, and *Lauraceae*) had IVs greater than 2% in Bawangling, Diaoluoshan, and Jianfengling, respectively.

**TABLE 2:** Phylogenetic structure of the *D. pectinatum* community at the three sites

Site	Number of plots (percentage, %)				Mean	
	NRI > 0	NRI < 0	NTI > 0	NTI < 0	NRI	NTI
BWL	20 (66.67)	10 (33.33)	15 (50.00)	15 (50.00)	0.41	0.07
DLS	1 (12.50)	7 (87.50)	0 (0.00)	8 (100.00)	−1.35	−0.90
JFL	13 (43.33)	17 (56.67)	5 (16.67)	25 (83.33)	−0.50	−0.70

Note

BWL, DLS, and JFL represent Bawangling, Diaoluoshan, and Jianfengling, respectively.

### 2.3 Environmental factor measurement

Soil samples were randomly taken along the diagonal (upper left, middle, and lower right) in each of the 30 × 30 m plots. A core of the top 20 cm of soil was removed at each point. Soil samples were mixed into a single sample and dried in the laboratory. After sieving, the physical and chemical indicators of the soil samples were measured in accordance with international standard methods. The indicators included the soil organic matter (SOM, g/kg, potassium dichromate oxidation volumetric method, LY/T 1237‐1999), soil total nitrogen (TN, g/kg, Kjeldahl boiling‐diffusion method, LY/T 1228‐1999), soil total phosphorus (TP, g/kg, sodium hydroxide alkaline fusion‐molybdenum anticolorimetric method, LY/T 1232‐1999), soil alkaline nitrogen (AN, mg/kg, alkaline hydrolysis‐ diffusion method, LY/T 1229‐1999), soil available phosphorus (AP, mg/kg, 0.05 mol/L HCl‐0.025 mol/L 1/2 H_2_SO_4_ extraction method, LY/T 1233‐1999), and soil pH (pH, H_2_O 1:2.5 potentiometry, LY/T 1239‐1999). In each 30 × 30 m plot, elevation (EL, m), latitude, and longitude data were collected with a global positioning navigation system (GPS, model: Geoxh6000, Trimble, USA; positioning accuracy: 10 cm + 1 cm). In addition, stand characteristics (e.g., canopy density, CD) and other topographic factors (e.g., aspect, AS; slope aspect, SP; and slope, SL) were also recorded. Note that canopy density refers to the ratio of the total projected area (crown width) of the tree crown on the ground under direct sunlight to the total area of forestland (stand). The canopy density was estimated by the mechanical spot method. In the stand survey, four sample points were arranged mechanically, and the surveyor looked up vertically at each position to judge whether the sample points were covered by the tree crown; if it was, the point was counted, and if not, they were not counted. Finally, the covered sample points were counted, and then, the canopy density was calculated. Canopy density = number of samples covered by tree crown/total number of samples (total crown width/total area of sample).

### 2.4 Diversity indices

#### 2.4.1 **Species α‐ and** β‐**diversity**

In this study, we used the species richness index (SR) as a measure of species α‐diversity, which represents the number of species in each plot. To analyze the patterns of the species β‐diversity along various environmental gradients, the Jaccard index was calculated using the vegan package in R version 3.5.3. The formula for this calculation is as follows (Jaccard, 1912):

(1) $$\mathrm{Jaccard}=\frac{\left(b+c\right)}{\left(a+b+c\right)}$$

Here, “*a*” represents the number of cooccurring species in two plots, and “*b*” and “*c*” represent the numbers of unique species in the two plots. An increase in the Jaccard index indicates that the difference in species compositions has increased.

#### 2.4.2 Phylogenetic tree construction

**FIGURE 3:**
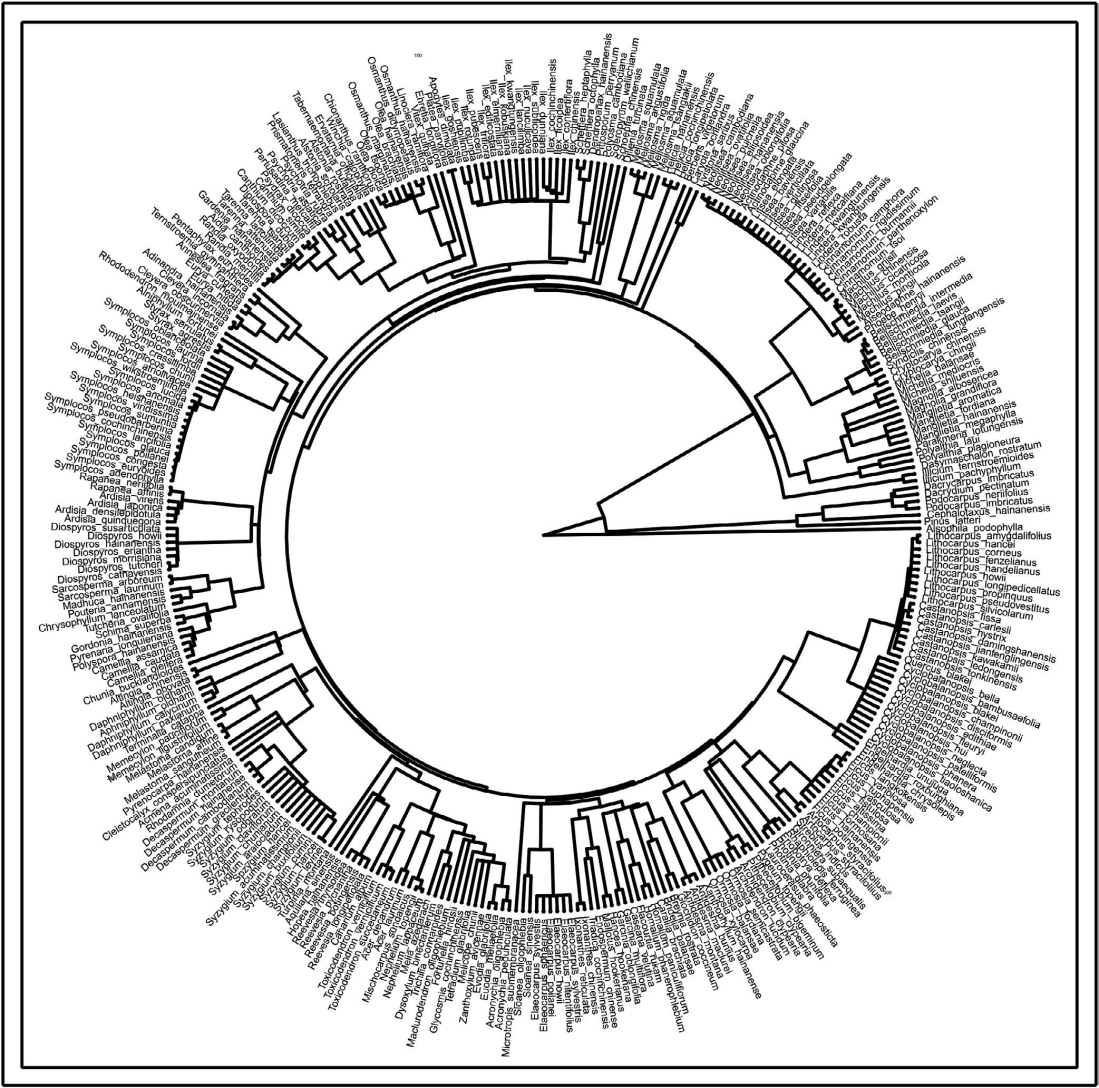
Phylogenetic tree of *Dacrydium pectinatum* communities constructed using V. PhyloMaker, including 344 taxa distributed across 150 genera in 69 families

The phylogenetic supertree should be established before phylogenetic diversity analysis. Hence, we used V. PhyloMaker (a freely available package for R) (Jin & Qian, 2019) and the woody plant list (which includes 346 species, 150 genera, and 69 families) created from the comprehensive field surveys performed at the three sites for supertree construction. V. PhyloMaker provides an inclusive species‐level time‐calibrated mega‐phylogeny for seed plants, and this mega‐phylogeny was used as a backbone to build the phylogenetic supertree (Figure 3). This procedure shifted the phylogenetic data of the analyses to an optimum level.

#### 2.4.3 **Phylogenetic α‐ and β**‐**diversity**

The statistical analyses and data processing were mainly carried out in the statistical software R 3.6.3. Before calculating phylogenetic diversity, it is necessary to pair species data and the phylogenetic tree. The picante package was used to detect whether the species names in the two datasets matched, and the datasets were reordered to replace the original data with the filtered combined data. Faith's PD (PD) was used as a measure to describe the phylogenetic α‐diversity, and this value is the sum of the minimum spanning path in the phylogenetic tree connecting all species found in a local plot (Faith, 1992). In recent years, various phylogenetic β‐diversity measures have been proposed, such as the mean phylogenetic dissimilarity (*D_pw_*) between the individuals or species in two communities (Rao, 1982; Webb et al., 2008), the mean nearest taxon distance (*D_nn_*) between the individuals or species in two communities (Webb et al., 2002, 2008), and the amount of phylogenetic branch length shared between species in two communities (Bryant et al., 2008). We used the mean nearest taxon distance (*D_nn_*) to assess the phylogenetic β‐diversity in this manuscript, and the calculation formula is as follows:

(2) $$D_{\mathrm{nn}}=\frac{\sum_{i=1}^{{\mathrm{nk}}_{1}}{\min\delta }_{{\mathrm{ik}}_{2}}+\;\sum_{j=1}^{{\mathrm{nk}}_{2}}{\min\delta }_{{\mathrm{jk}}_{1}}}{2}$$

Here, ${\min\delta }_{{\mathrm{ik}}_{2}}$ represents the nearest phylogenetic distance between species *i* in community *k*_1_ and species in community *k*_2_, and ${\min\delta }_{{\mathrm{jk}}_{1}}$ represents the nearest phylogenetic distance between species *j* in community *k*_2_ and species in community *k*_1_.

The net relatedness index (NRI) and the nearest taxon index (NTI) are used to quantify and describe the phylogenetic structures in different communities (Webb, 2000). In general, when the species pool involved in the analysis is large, the combination of the NTI and NRI effectively reflects the assembly mechanism of the community (Kraft et al., 2008). The NRI refers to the standardized effect size of the mean phylogenetic distance (MPD), which measures the mean phylogenetic distance between each of the sampled taxa and every other terminal in the sample. The NTI is related to the mean nearest phylogenetic taxon distance (MNTD), which measures the mean distance between each of the sampled taxa and its own most closely related terminal taxon in the sample (Molina‐Venegas & Roquet, 2013). Before calculating the NRI and NTI, it was first assumed that all the species surveyed in the community constituted a local species pool. Keeping the total number of species unchanged, the species in each plot was randomly selected 999 times from the species pool through the random lottery model to obtain the MPD/MNTD distribution of the species in each plot under the random null model. Finally, the random distribution results were used to standardize the observations of MPD/MNTD to obtain the NRI and NTI. The formulas for the calculation of the NRI/NTI are as follows:

(3) $$\mathrm{NRI}=‐1\times \frac{\left({\mathrm{MPD}}_{\mathrm{observed}}‐{\mathrm{MPD}}_{\mathrm{randomized}}\right)}{SD({\mathrm{MPD}}_{\mathrm{randomized}})}$$

(4) $$\mathrm{NRI}=‐1\times \frac{\left({\mathrm{MNTD}}_{\mathrm{observed}}‐{\mathrm{MNTD}}_{\mathrm{randomized}}\right)}{SD({\mathrm{MNTD}}_{\mathrm{randomized}})}$$

MPD_observed_ and MNTD_observed_ are the actual values of the MPD and MNTD, respectively. MPD_randomized_ and MNTD_randomized_ are the means of the null model distributions (*n* = 999). sdMPD_randomized_ and sdMNTD_randomized_ are the standard deviations of the null model distributions.

If NRI/NTI > 0, MPD/MNTD is lower than the expected value, indicating phylogenetic structure convergence, which means that the species within a community are less closely related than expected; in contrast, NRI/NTI < 0 indicates phylogenetic structure overdispersion, which means that the species within a community are more distantly related than expected by chance. The analyses were performed using the picante package in R version 3.5.3 (Kembel et al., 2010).

### 2.5 Data analysis

We wanted to understand how the species and phylogenetic α‐diversity shift along environmental gradients. We compared the α‐diversity indices (SR, PD, NRI, and NTI) across the three study areas by ANOVA. If the variation was significant (*p* < .05), we performed multiple comparisons using Tukey's honest significant differences (HSD) to determine which sites differed significantly. In addition, we performed linear or nonlinear regression analysis to detect the relationships between α‐diversity and elevation. The species and phylogenetic α‐diversity indices were used as response variables separately, while elevation was used as an explanatory variable.

Correlations between species and phylogenetic α‐diversity and environmental factors were assessed by using stepwise multiple regression. SR and PD were used as response variables separately, while the explanatory variables included soil factors (SOM, PH, TN, TP, AN, and AP), topographic factors (EL, SL, and AS), and forest stand factors (CD). In both models, all explanatory variables were standardized by subtracting the mean and dividing by standard deviation prior to modeling. Then, we performed a multicollinearity test on the explanatory variables (variance inflation factor, VIF, which is a measure of the severity of multicollinearity in a multiple linear regression model). The test results showed that the collinear degree between all variables was weak (VIF < 5), which allows further analysis. The analysis was performed by the R package lme4 (Bates et al., 2014). At each step, one variable was added to the regression equation. The added variable was the one that induced the greatest reduction in the error sum of squares. It was also the variable that had the highest partial correlation with the dependent variable for fixed values of those variables already added (Jiang et al., 2015). The selection of the best model depends on the difference between *R* ^2^ and the model's Akaike information criterion (AIC) values. The relative importance of the impact of environmental factors on dependent variables was evaluated in the selected model by the mean of the path coefficients, and a high path coefficient indicates a strong effect. Finally, the results of the regression model retained only the environmental variables with *p* < .05. The analyses were performed in R version 3.6.3.

To explore the change in species and phylogenetic β‐diversity among communities, we analyzed the relationship between β‐diversity and environmental distance and geographical distance. Environmental distances were estimated by calculating the Euclidean distances between plots based on their precipitation and elevation. The precipitation data originated from the China Meteorological Data Network of the China Meteorological Administration Meteorological Data Center http://data.cma.cn/en, and the data for each plot were calculated by kriging interpolation. This analysis was performed by ArcGIS 10.6. The geographical distance between each pair of plots was calculated using GPS coordinates taken from each plot and the R package fossil (Zhang et al., 2013). We combined the distances of precipitation and elevation as a representative matrix of environmental heterogeneity and subsequently partitioned the variance. We used Mantel's test to quantify the correlation between the species and phylogenetic dissimilarity matrix, geographical distance, and environmental distance (Zhang et al., 2013). The analyses were performed using the ecodist package in R version 3.6.3. Each Mantel's test generated an *r* value similar to Pearson's correlation index, which represents the correlation between the distance matrices. Permutation tests were applied to assess the significance of the correlation by randomizing the distance matrix 999 times. We also used the vegan package and analyzed the independent interpretations of the environmental distance and geographical distance relative to the β‐diversity using variance decomposition.

## 3 RESULTS

### 3.1 Variations in species and phylogenetic α‐diversity of the *Dacrydium pectinatum* community at local and regional scales

**FIGURE 4:**
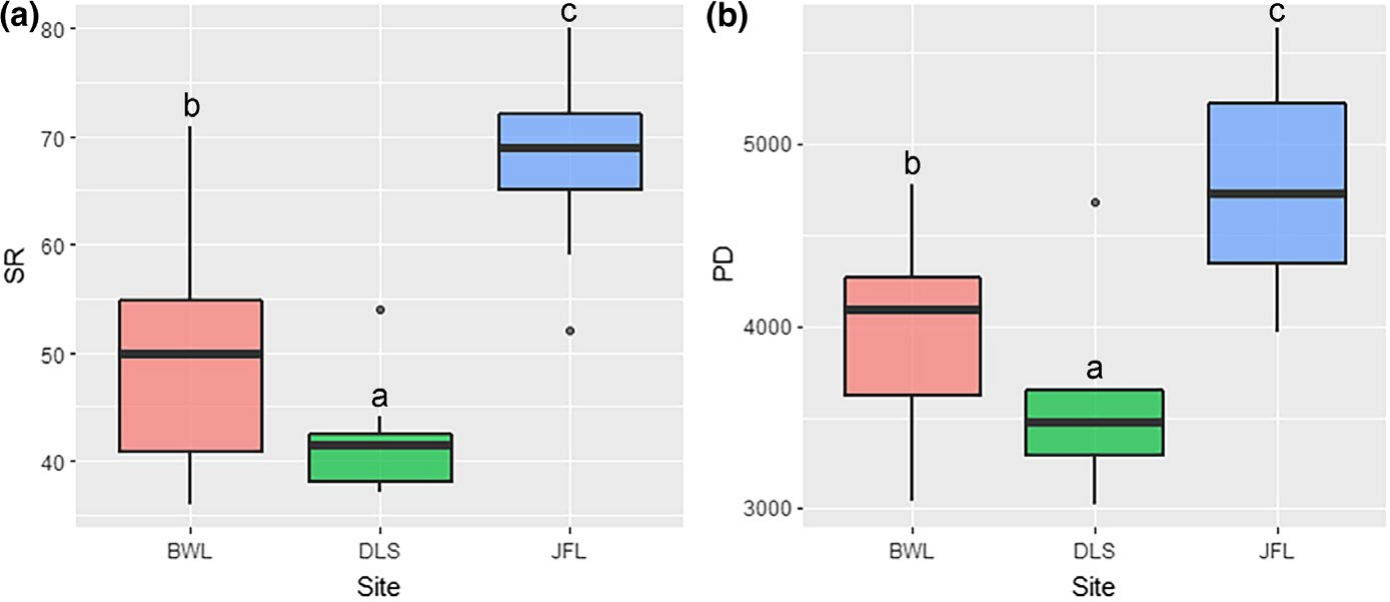
Tukey's HSD analysis showed that the species and phylogeny α‐diversity changed at the local scale. BWL, DLS, and JFL represent Bawangling, Diaoluoshan, and Jianfengling, respectively. Panel a represents species α‐diversity (SR); panel b represents phylogenetic α‐diversity (PD). Data with different letters are significantly different at *p* < .05. Note that the black dotted lines in panels c and d represent completely random values

On the local scale, the three sites present different α‐diversity patterns (Figure 4a,b). The maximum values of SR and PD appeared in Jianfengling (elevation between 830 and 1,053 m) and showed significant differences from the other sites in terms of diversity (*p* < .05), which was related to the higher species richness of Jianfengling. Bawangling (elevation between 1,062 and 1,300 m) and Diaoluoshan (elevation between 916 and 986 m) have relatively low species and phylogenetic diversity, and the minimum values of SR and PD appear in Diaoluoshan. The phylogenetic structure of the three sites also showed different patterns (Table 2). For Bawangling, the mean NRI and NTI were 0.41 and 0.07, respectively; the NRI of 20 (66.67%) plots and NTI of 15 (50%) plots were positive, indicating that the phylogenetic structure of most communities presented convergence patterns. For Diaoluoshan, the mean NRI and NTI were −1.35 and −0.90, respectively; only one plot had a positive NRI, and the rest were negative, indicating that the phylogenetic structure presented an overdispersed pattern. For Jianfengling, the mean NRI and NTI were −0.50 and −0.70, respectively; the NRI of 17 (56.67%) plots and NTI of 25 (83.33%) plots were negative, indicating that the phylogenetic structure of most communities presented an overdispersed pattern.

**FIGURE 5:**
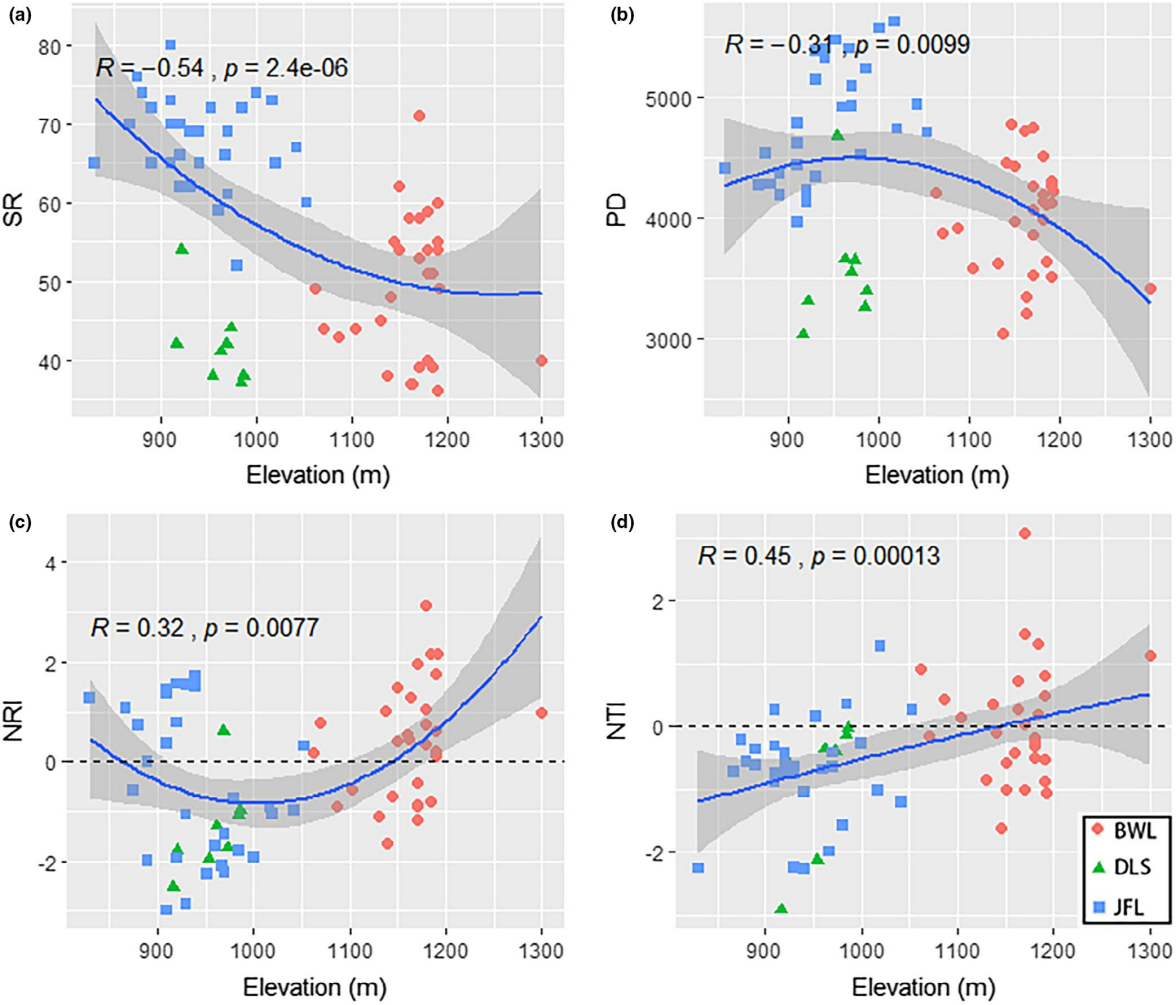
LOESS regression analysis showed that species and phylogenetic α‐diversity had different elevation patterns at the community scale. The blue dots represent Bawangling (BWL), the green dots represent Diaoluoshan (DLS), and the red dots represent Jianfengling (JFL). Panel a represents species α‐diversity (SR); panel b represents phylogenetic α‐diversity (PD); panel c represents relatedness indices (NRI); and panel d represents nearest taxon indices (NTI). Note that the black dotted lines in panels c and d represent completely random values

On the regional scale, the species and phylogenetic α‐diversity decreased from high elevation to low elevation (Figure 5a,b). However, the difference was that SR presents a monotonic decreasing pattern with elevation, and PD shows a unimodal relationship with elevation. As elevation increases, PD first increases and then decreases, and the peak appears between 900 m and 1,000 m. The phylogenetic structure of the community increased with elevation (Figure 5c,d). The NRI and NTI of most plots in low‐elevation areas are negative. However, in high‐elevation areas, the NRI and NTI of most plots are positive. The results showed that the phylogenetic structure of the community changed from overdispersed to convergent with increasing elevation.

### 3.2 Changes in species and phylogenetic β‐diversity of the *Dacrydium pectinatum* community along various environmental gradients

**FIGURE 6:**
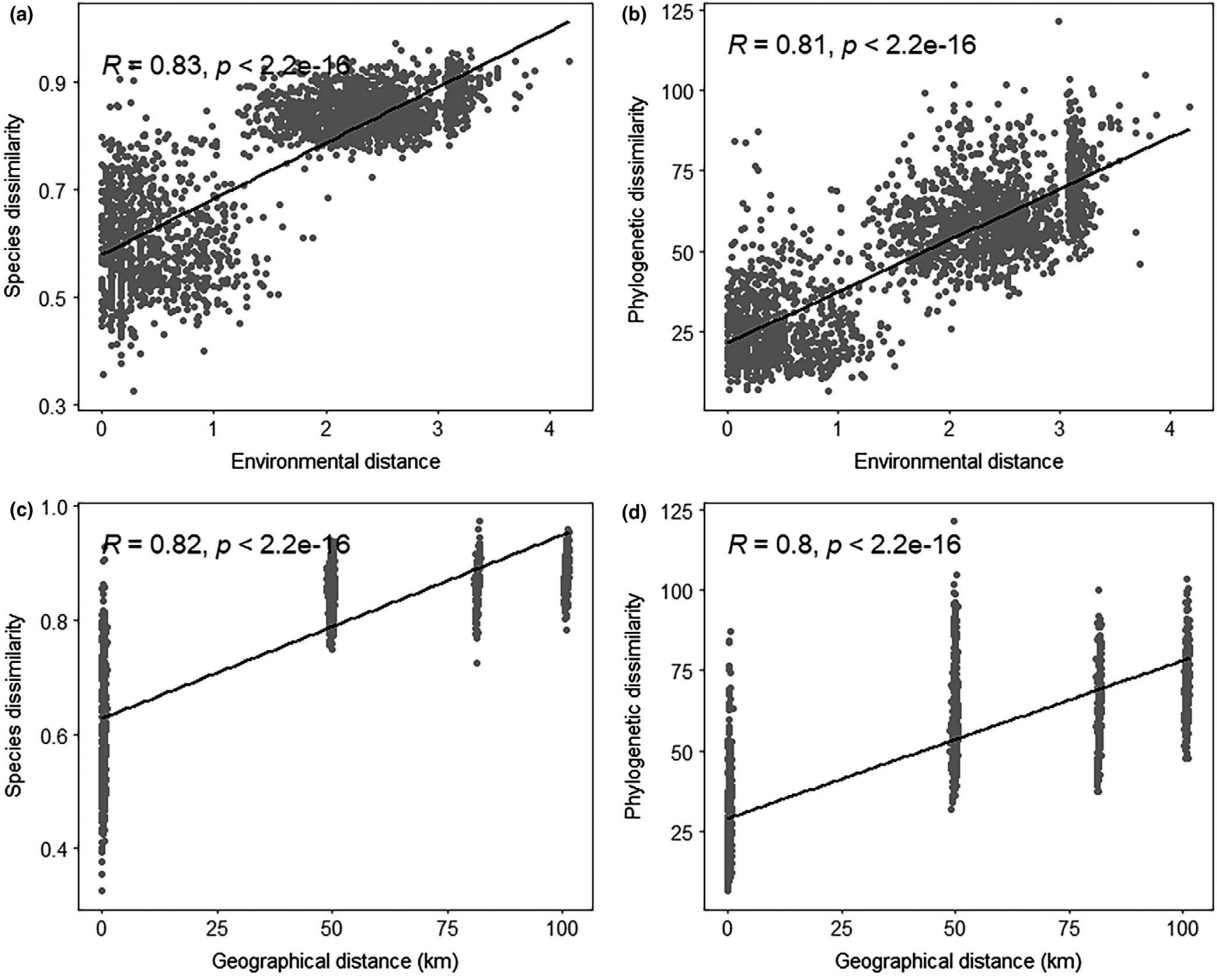
Species and phylogenetic β‐diversity shifts with environmental and geographical distance. Panels a and c illustrate the linear relationships between the species β‐diversity and environmental distance, respectively. Panels b and d illustrate the linear relationships between phylogenetic β‐diversity and geographical distance, respectively

Species and phylogenetic β‐diversity showed a consistent pattern (Figure 6); with increasing environmental distance and geographical distance, the β‐diversity among communities increased; that is, the species turnover rate within the community increased with increasing environmental and geographical distance between plots. This result indicates that the change in species and phylogenetic β‐diversity may be jointly affected by environmental filtering and dispersal‐limiting effects.

### 3.3 Main factors affecting the species and phylogenetic diversity patterns of the *Dacrydium pectinatum* community

**FIGURE 7:**
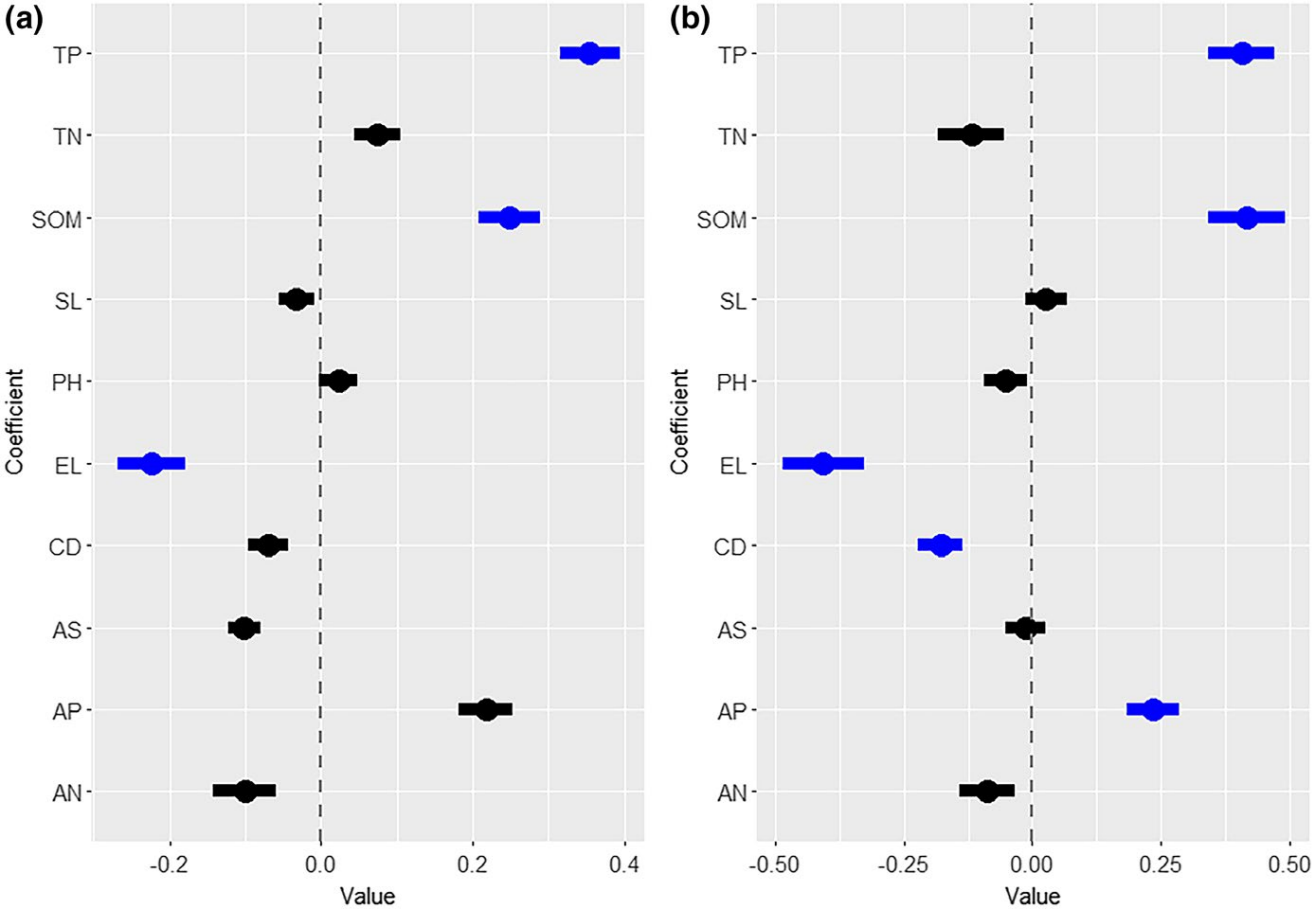
Effects of environmental factors on community‐level species and phylogenetic α‐diversity. Panels a and b show the coefficients (with 95% confidence intervals) of the regressions between various environmental factors and SR and PD, respectively. Blue dots indicate statistical significance (*p* < .05), and black dots indicate no statistical significance (*p* > .05). Data for the soil total phosphorus (TP, g/kg), soil total nitrogen (TN, g/kg), soil organic matter (SOM, g/kg), slope (SL, °), soil pH (PH), elevation (EL, m), canopy density (CD, %), aspect (AS), soil available phosphorus (AP, mg/kg), and soil available nitrogen (AN, mg/kg) are provided

We used two multiple stepwise regression models to filter out the most important environmental factors that affect the community‐level species and phylogenetic α‐diversity, and the variance explanation percentages for PD and SR were 48.8% and 64.9%, respectively (Table 3). PD was significantly correlated with increased TP and SOM (Figure 7a). The SR showed a similar trend, significantly increasing with increasing TP, SOM, and AP but significantly decreasing with increasing EL and CD (Figure 7b).

**TABLE 3:** Stepwise multiple regression between the community‐level species and phylogenetic α‐diversity and various environmental variables

Environmental variables	Regression coefficients	Parameters			
		*T*	*p*	*R* ^2^	*AIC*
PD				0.468	−40.30
Soil organic matter	0.249	3.077	.008**		
Soil total phosphorus	0.708	6.703	.000**		
Elevation	−0.224	2.490	.040*		
SR				0.649	−65.54
Soil organic matter	0.417	4.226	.000***		
Soil total phosphorus	0.408	4.065	.000***		
Soil available phosphorus	0.234	2.453	.017*		
Elevation	−0.407	−2.930	.005**		
Canopy density	−0.179	−2.273	.027*		

Note that only the environmental variables with statistical significance were retained in the regression model (*p* < .05).

* *p* < .05.

** *p* < .01.

*** *p* < .001.

**FIGURE 8:**
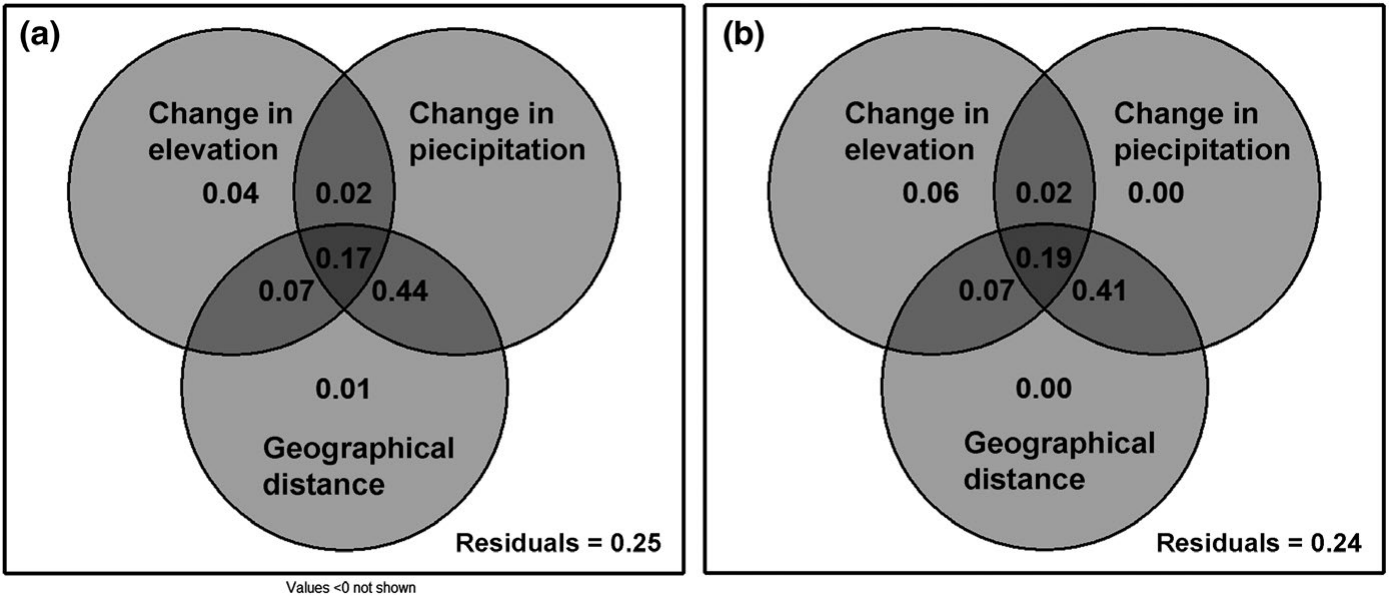
Variance in the species and phylogenetic β‐diversity of the *Dacrydium pectinatum* community explained by environmental distance and geographical distance. Panel a represents the species β‐diversity, and panel b represents the phylogenetic β‐diversity

Elevation distance and geographical distance had a significant impact on the change in species β‐diversity (Figure 8a). However, elevation, which represents environmental heterogeneity, has a higher variance explanation rate for species β‐diversity differences than geographical distance, which indicates that environmental differences were more sensitive to changes in species β‐diversity. Elevation, precipitation, and geographical distance all affected the changes in phylogenetic β‐diversity (Figure 8b), which was consistent with the pattern of species β‐diversity. The role of elevation was relatively strong, while the roles of geographical distance and precipitation were relatively weak.

## 4 DISCUSSION

### 4.1 Species and phylogenetic α‐diversity patterns of the *Dacrydium pectinatum* community change across elevation gradients and at the local scale

The community‐level α‐diversity patterns of the *D. pectinatum* community were consistent in the species‐based and phylogenetic‐based analyses in our study. Both presented a monotonically decreasing pattern with elevation, with the peak occurring in the mid‐elevation region between 800 and 1,000 m (Figure 5). Our study confirms previous assumptions. Rahbek (2004) indicated that species distributions might show a moderate expansion phenomenon in mid‐elevation regions due to a greater abundance of available resources and the presence of the most suitable hydrothermal conditions. Conversely, some harsh environmental conditions in high‐elevation regions, such as low temperatures, a high frequency of fog, and scarce soil nutrients, will cause some species present at low elevations to be filtered out with increasing elevation (Guevara, 2005). In addition, the changes in α‐diversity at the local scale also indirectly validated the environmental filtering hypothesis; for instance, Jianfengling in mid‐elevation areas was significantly more diverse than Bawangling and Diaoluoshan due to the more suitable hydrothermal conditions (Figure 4). This evidence indicates that environmental filtering plays an important role in the formation of species and phylogenetic α‐diversity. We also found that although Jianfengling and Diaoluoshan had similar habitat conditions, they had different diversity patterns. We believe that this difference might be explained by the time‐species formation hypothesis (Lomolino, 2001) because regions with earlier colonization histories generally tend to have higher diversities (note Jianfengling and Diaoluoshan nature reserves were established in 1960 and 1984, respectively).

The phylogenetic structure of a plant community can reveal the ecological process of diversity distribution (Kembel & Hubbell, 2006). The phylogenetic structure of the *D. pectinatum* community essentially showed an overdispersion pattern in low‐elevation regions, but a convergence pattern appeared in some relatively high‐elevation plots (Figure 5). A previous study indicated that the phylogenetic structure of a tropical rainforest community in Malaysia presented an overdispersion pattern at low elevations and shifted to a convergence distribution pattern at high elevations (Webb, 2000). Huang et al. (2010) found a similar pattern in a subtropical evergreen broad‐leaved forest. Based on the differences across the three study sites, we found that Diaoluoshan and Jianfengling both showed phylogenetic overdispersion patterns at low‐middle elevations, while some plots at Bawangling at higher elevations showed a convergent distribution (Table 2). Hence, environmental filtering might cause the phylogenetic structure of the community to tend to converge in relatively high‐elevation regions, which in turn affects the assembly patterns of the plant community (Souza‐Neto et al., 2015). However, in low‐ and mid‐elevation regions, community assembly is mainly affected by various factors, such as environmental filtering, similarity restrictions, and anthropogenic disturbance (Kraft et al., 2008). For example, Ding et al. (2011) suggested that in tropical forest habitats with high species richness and some degree of disturbance, phylogenetic structures are most likely to show an overdispersed pattern.

In our study, elevation had a significant effect on the species and phylogenetic α‐diversity (Figure 7 and Table 3). Changes in elevation across a relatively short geographical distance result in large environmental changes in factors such as climate, geometric constraints (e.g., boundary constraints), and anthropogenic activity intensity (Zhang et al., 2015). Hence, elevation usually indirectly affects the assembly patterns of plant communities by changing other environmental factors (Rahbek, 2004). The species and phylogenetic α‐diversity showed a significant positive correlation with the SOM, TP, and AP and a negative correlation with the canopy density in this study (Figure 7 and Table 3). Organic matter contains nutrients necessary for plant growth, and its absence will lead to a reduction in the number of species, which in turn will cause a decline in species diversity (Fornara et al., 2009). Previous studies have found that phosphorus is generally lacking in tropical forest soils (note that the total phosphorus content is mostly below 0.8 g/kg) (Cleveland et al., 2011). Phosphorus is a key component of photosynthesis in plants, and a lack of this nutrient will affect diversity distribution patterns (Long et al., 2011). The canopy density directly affects the ability of the vegetation under the canopy to obtain light, which in turn affects the natural regeneration of the community (Gao et al., 2017). Many reports have indicated that light is the main source of energy required for the growth of tree seedlings and saplings, and thus, canopy density usually plays an important role in the coexistence of species in tropical forests (Jiang et al., 2015).

### 4.2 Changes in the species and phylogenetic β‐diversity patterns of the *Dacrydium pectinatum* community along environmental gradients

In general, β‐ diversity is used to assess the mechanisms of community assembly in heterogeneous spaces by exploring changes in species compositions, phylogenetic structures, and functional traits among communities (Cardoso et al., 2014). The greater the differences in species similarity, phylogenetic relationships, and trait combinations along environmental gradients, the higher the β‐diversity typically is among communities (Legendre, 2007). In this study, the species and phylogenetic β‐diversity of the *D. pectinatum* community showed consistent positive correlations with environmental distance and geographical distance (Figure 6). In addition, with changes in various distances, the differences in species composition between communities increased, which indicates that environmental filtering and dispersal restrictions affect the turnover of plants in the community. Under the assumption of the Rapoport hypothesis, plants occupy little space and resources in tropical forests, usually exhibiting environmental specificity, and show a high species turnover rate, even at the spatial scale with the fewest changes (Simberloff et al., 1983). Previous studies have suggested that both deterministic and stochastic processes play a role in plant community assembly in tropical and subtropical environments and that the coexistence and maintenance of species are the result of random drift and niche differentiation (Muñoz et al., 2016). For example, Lu et al. (2013) found that species β‐diversity is significantly affected by distance and topography and that its correlations with geographical distance and topographic differences show similar monotonic increasing trends with increasing differences. Zhang et al. (2012) also revealed similar results, showing that changes in phylogenetic β‐diversity are related to geographical distance and environmental differences and that the phylogenetic structure changes from overdispersion to convergence with increasing spatial scales.

However, the relative contributions of deterministic and stochastic processes to community assembly patterns at the spatial scale—that is, the identification of which factors play key roles—remain controversial. One view is that the main factors affecting the species compositions of communities are two opposite processes, habitat filtering and similarity limitation (Webb et al., 2002), and another is that the differences in composition between communities are related only to geographical distance and not to other environmental factors as the spatial scale increases (Chase & Myers, 2011). In fact, the relative importance of these processes varies across different habitats and scales. For example, in grassland communities, the community structure is relatively simple, and niche differentiation thus plays a major role, while in species‐rich tropical forests, the maintenance of species diversity is mainly dominated by neutral processes (Gravel et al., 2006). Based on the variance results, environmental differences and geographical distances have an impact on changes in species and phylogenetic β‐diversity, but the impact of environmental differences is stronger than that of geographical distance (Figure 8), which indicates that environmental filtering plays a more key role than dispersal restriction in driving the assembly of tropical plant communities in a heterogeneous space with complex habitats.

## 5 CONCLUSION

Our study found that the species and phylogenetic α‐diversity patterns of the *D. pectinatum* community showed differences at a local scale, which may be related to the local colonization history and the changes in environmental factors. On a regional scale, two α‐diversity pattern variations converged, and they decreased as the elevation increased and showed a close relationship with topography, soil, and stand factors. The phylogenetic structure changes from overdispersion to convergence with increasing elevation, which indicates the environmental filtering effect of high‐elevation areas. Both environmental distance and geographical distance have a significant impact on species and phylogenetic β‐diversity variation, but the relative effect of environmental differences is greater than that of geographical distance, indicating that environmental filtering is the key factor affecting β‐diversity patterns. Combining species‐based and phylogenetic‐based methods strongly proves the key role played by the habitat filtering hypothesis in the assembly process of the *D. pectinatum* community. Of course, it is necessary to combine more diversity patterns to explore the change mechanism of plant diversity on Hainan Island, which will help address the potential threat of biodiversity loss.

## CONFLICT OF INTEREST

None declared.

## AUTHOR CONTRIBUTIONS

**Haodong Liu:** Data curation (lead); formal analysis (lead); investigation (lead); methodology (equal); software (lead); writing–original draft (lead); writing–review and editing (lead). **Hua Liu:** Methodology (equal). **Yongfu Chen:** Methodology (supporting); project administration (supporting); resources (equal). **Zhiyang Xu:** Methodology (equal); software (equal). **Yunchuan Dai:** Software (equal). **Qiao Chen:** Conceptualization (lead); funding acquisition (lead); methodology (lead); resources (lead); writing–review and editing (equal). **Yongkang Ma:** Investigation (equal); software (equal).

## Supporting information

Appendix S1

Fig S3

## Data Availability

All authors have agreed to deposit data from this manuscript to a public repository. The data have been submitted to Dryad, and the DOI number is https://doi.org/10.5061/dryad.pc866t1mj.
