# Supplementary material for: Identifying the patterns of changes in α‐ and β‐diversity across Dacrydium pectinatum communities in Hainan Island, China
Source: Ecol Evol. 2021 Mar 13;11(9):4616–30. doi: 10.1002/ece3.7361 (PMC8093751; doi:10.1002/ece3.7361)
Supplement: Supplementary file 1 — Appendix S1 [file ECE3-11-4616-s002.docx]

**Appendix S1**

**Table S1** Summary of the plant species composition and stand characteristics of the *D. pectinatum* community.

| Site | No. of  families | No. of  genera | No. of  species | No. of  stems | DBH | H | N |
| --- | --- | --- | --- | --- | --- | --- | --- |
| BWL | 52 | 101 | 187 | 5386 | 14.51±1.34^a^ | 10.41±0.74^a^ | 1994.43±511.65^b^ |
| DLS | 41 | 73 | 126 | 1023 | 14.35±8.68^a^ | 10.44±1.29^a^ | 1700.93±409.84^a^ |
| JFL | 54 | 110 | 186 | 5705 | 15.15±1.11^b^ | 10.73±1.39^b^ | 2112.59±357.39^c^ |

Species data are based on tree abundance data from sixty-eight permanent plots in *D. pectinatum* communities*.* BWL represents Bawangling, DLS represents Diaoluoshan, and JFL represents Jianfengling. The mean diameter at breast height of the stand (DBH, cm), mean height of the stand (H, m), and density of the stand (N, stems/hm^2^) are expressed as the mean ± sd.

**Table S2** Dominance of 30 species selected using calculated importance values (IV = relative frequency + relative density + relative dominance) across the three *D. pectinatum* communities.

| Species | Family | IV | N | H | H_max_ | DBH | D_max_ | BA |
| --- | --- | --- | --- | --- | --- | --- | --- | --- |
| Bawangling |  |  |  |  |  |  |  |  |
| *Dacrydium pectinatum* | *Podocarpaceae* | 8.68 | 34 | 18.2 | 30.0 | 59.5 | 194.0 | 13.78 |
| *Castanopsis carlesii* | *Fagaceae* | 4.94 | 127 | 12.6 | 22.6 | 17.4 | 68.8 | 4.05 |
| *Syzygium araiocladum* | *Myrtaceae* | 4.89 | 167 | 9.8 | 20.8 | 12.5 | 33.7 | 2.49 |
| *Cryptocarya chinensis* | *Lauraceae* | 3.27 | 129 | 7.8 | 17.0 | 9.5 | 31.1 | 1.16 |
| *Illicium ternstroemioides* | *Magnoliaceae* | 3.01 | 111 | 8.0 | 18.1 | 9.4 | 27.4 | 0.88 |
| *Xanthophyllum hainanense* | *Polygalaceae* | 2.70 | 59 | 11.9 | 21.2 | 16.8 | 53.0 | 1.82 |
| *Ilex kobuskiana* | *Aquifoliaceae* | 1.95 | 56 | 9.8 | 19.0 | 11.6 | 29.3 | 0.72 |
| *Syzygium championii* | *Myrtaceae* | 1.80 | 49 | 9.3 | 20.0 | 11.0 | 88.0 | 0.75 |
| *Symplocos adenophylla* | *Symplocaceae* | 1.49 | 47 | 9.1 | 19.7 | 8.6 | 14.5 | 0.30 |
| *Lindera robusta* | *Lauraceae* | 1.44 | 41 | 11.1 | 17.0 | 12.2 | 36.9 | 0.59 |
| Diaoluoshan |  |  |  |  |  |  |  |  |
| *Dacrydium pectinatum* | *Podocarpaceae* | 15.86 | 124 | 11.2 | 21.0 | 30.5 | 130.5 | 12.33 |
| *Cyclobalanopsis champinonii* | *Fagaceae* | 4.74 | 43 | 11.2 | 17.6 | 30.6 | 78.7 | 3.17 |
| *Altingia chinensis* | *Hamamelidaceae* | 3.00 | 39 | 11.3 | 18.0 | 22.2 | 50.7 | 1.51 |
| *Adinandra hainanensis* | *Theaceae* | 2.65 | 42 | 10.0 | 15.7 | 16.8 | 38.9 | 0.89 |
| *Michelia shiluensis* | *Magnoliaceae* | 2.59 | 46 | 10.0 | 16.4 | 15.9 | 39.5 | 0.83 |
| *Castanopsis carlesii* | *Fagaceae* | 2.54 | 42 | 9.6 | 15.6 | 18.2 | 38.2 | 0.98 |
| *Xanthophyllum hainanense* | *Polygalaceae* | 2.44 | 39 | 10.4 | 14.8 | 17.4 | 47.4 | 0.85 |
| *Castanopsis jianfenglingensis* | *Fagaceae* | 2.21 | 39 | 9.1 | 14.6 | 16.7 | 48.9 | 0.82 |
| *Alstonia scholaris* | *Apocynaceae* | 1.93 | 36 | 9.0 | 13.3 | 12.1 | 35.8 | 0.39 |
| *Daphniphyllum paxianum* | *Daphniphyllaceae* | 1.85 | 38 | 9.3 | 14.5 | 11.9 | 30.4 | 0.38 |
| Jianfengling |  |  |  |  |  |  |  |  |
| *Gironniera subaequalis* | *Ulmaceae* | 4.55 | 130 | 10.8 | 25.0 | 15.6 | 41.0 | 3.03 |
| *Livistona saribus* | *Palmae* | 4.48 | 67 | 8.9 | 28.0 | 31.2 | 50.0 | 5.28 |
| *Dacrydium pectinatum* | *Podocarpaceae* | 4.26 | 17 | 21.1 | 36.0 | 62.9 | 105.2 | 6.00 |
| *Cryptocarya chinensis* | *Lauraceae* | 3.74 | 123 | 10.2 | 30.0 | 11.7 | 35.8 | 1.70 |
| *Alseodaphne hainanensis* | *Lauraceae* | 3.00 | 69 | 10.8 | 25.0 | 17.1 | 55.8 | 2.23 |
| *Beilschmiedia tungfangensis* | *Lauraceae* | 2.35 | 64 | 10.6 | 30.0 | 12.1 | 43.5 | 1.13 |
| *Cryptocarya chingii* | *Lauraceae* | 2.06 | 63 | 9.7 | 22.0 | 10.1 | 25.9 | 0.60 |
| *Nephelium topengii* | *Sapindaceae* | 1.86 | 54 | 9.9 | 26.0 | 10.4 | 33.9 | 0.62 |
| *Psychotria rubra* | *Rubiaceae* | 1.60 | 49 | 5.6 | 11.0 | 7.1 | 13.7 | 0.21 |
| *Cinnamomum burmanni* | *Lauraceae* | 1.57 | 43 | 9.4 | 38.0 | 9.7 | 27.5 | 0.40 |

Species data from sixty-eight permanent plots of *D. pectinatum*. The abbreviations are as follows: mean diameter at breast height of the stand (DBH, cm), maximum diameter at breast height of the stand (D_max_, m), mean height of the stand (H, m), maximum height of the stand (H_max_, m), density of the stand (N, stems/hm^2^), and total basal area of the stand (BA, m^2^/hm^2^).


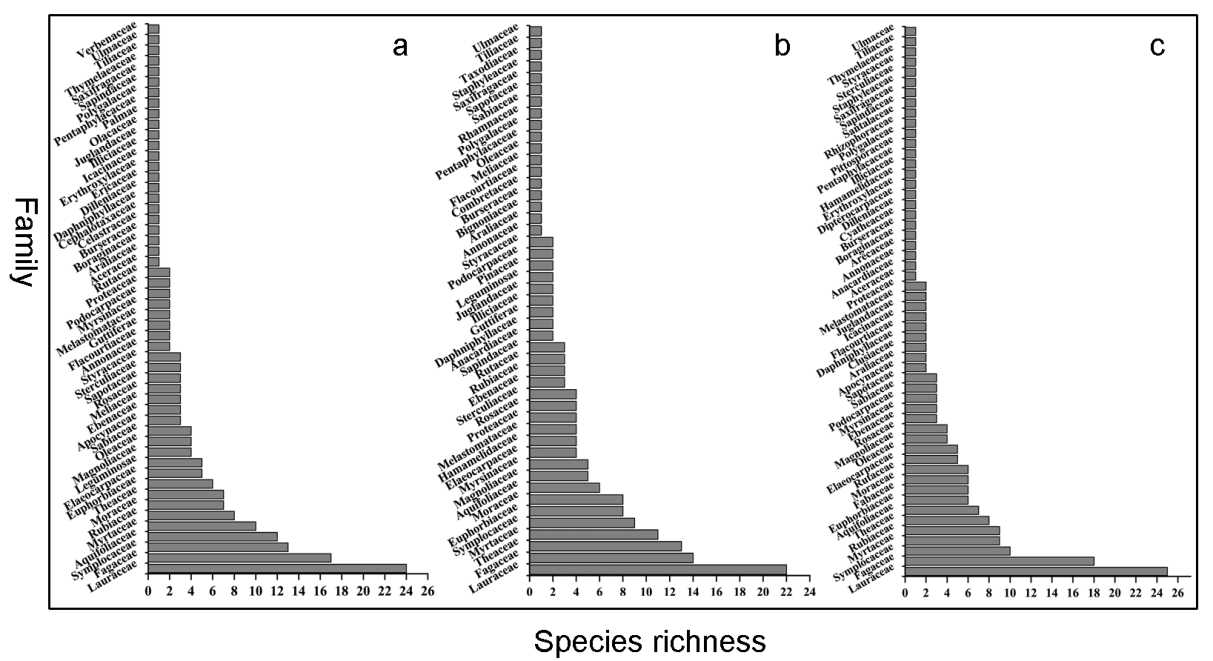


**Fig. S3** Abundance distribution of plant families in *D. pectinatum* communities. Panel a provides data for Bawangling, panel b provides data for Diaoluoshan, and panel c provides data for Jianfengling.
