# Supplementary figures and images for: Identifying the patterns of changes in α‐ and β‐diversity across Dacrydium pectinatum communities in Hainan Island, China
Source: Ecol Evol. 2021 Mar 13;11(9):4616–30. doi: 10.1002/ece3.7361 (PMC8093751; doi:10.1002/ece3.7361)

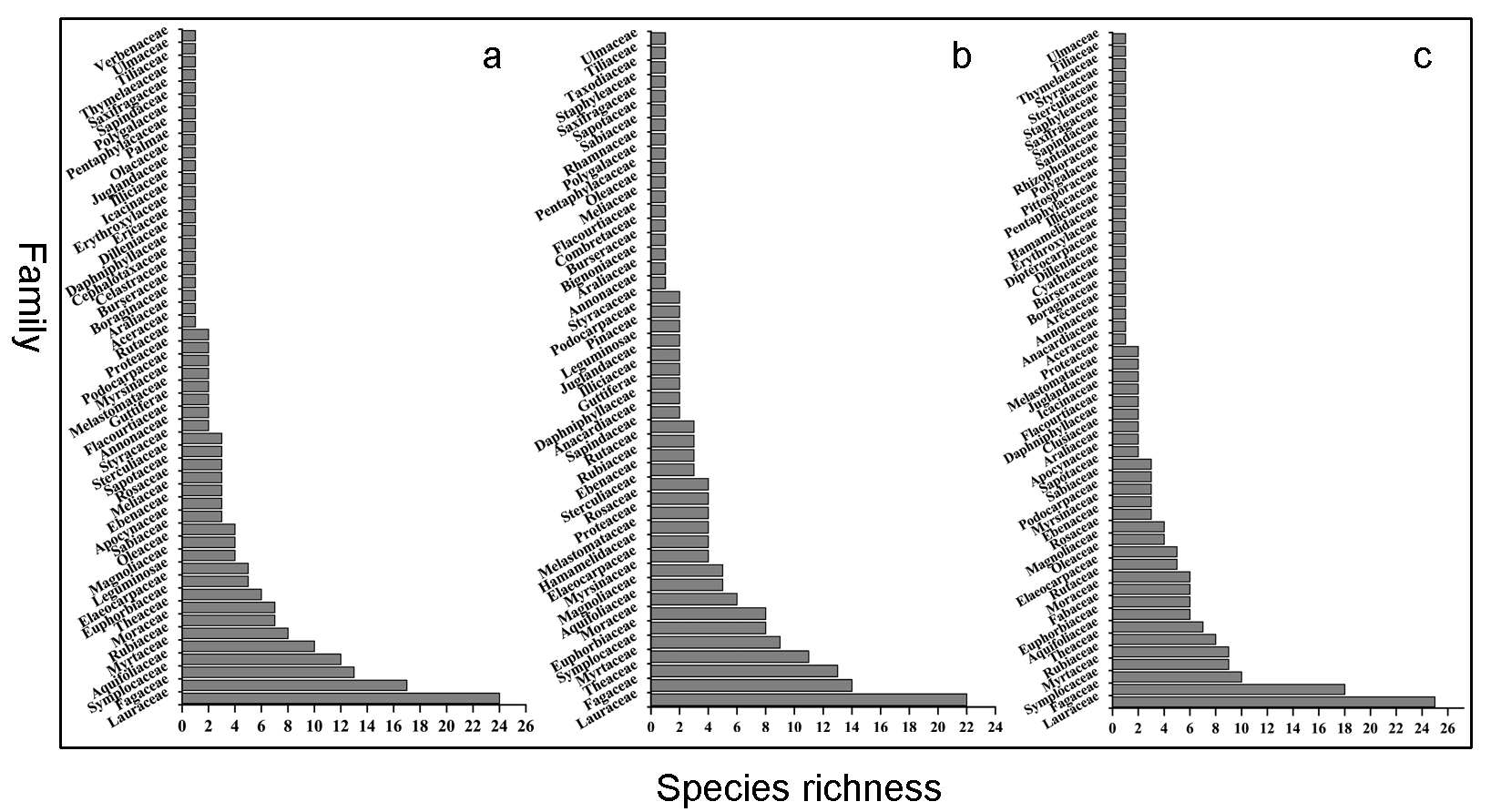

Supplement: Supplementary file 2 — Fig S3 [file ECE3-11-4616-s001.tif]
